# Highly Stretchable Hydrogels as Wearable and Implantable Sensors for Recording Physiological and Brain Neural Signals

**DOI:** 10.1002/advs.202201059

**Published:** 2022-03-31

**Authors:** Quanduo Liang, Xiangjiao Xia, Xiguang Sun, Dehai Yu, Xinrui Huang, Guanghong Han, Samuel M. Mugo, Wei Chen, Qiang Zhang

**Affiliations:** ^1^ State Key Laboratory of Electroanalytical Chemistry Changchun Institute of Applied Chemistry Chinese Academy of Sciences Changchun 130022 P. R. China; ^2^ School of Applied Chemistry and Engineering University of Science and Technology of China Hefei 230026 P. R. China; ^3^ Bethune First Hospital of Jilin University No. 1, Xinmin Street Changchun 130061 P. R. China; ^4^ Department of Oral Geriatrics Hospital of Stomatology Jilin University Changchun 130021 P. R. China; ^5^ Department of Physical Sciences MacEwan University Edmonton ABT5J4S2 Canada

**Keywords:** brain–machine interface, hydrogels, implantable sensors, microgels, wearable sensors

## Abstract

Recording electrophysiological information such as brain neural signals is of great importance in health monitoring and disease diagnosis. However, foreign body response and performance loss over time are major challenges stemming from the chemomechanical mismatch between sensors and tissues. Herein, microgels are utilized as large crosslinking centers in hydrogel networks to modulate the tradeoff between modulus and fatigue resistance/stretchability for producing hydrogels that closely match chemomechanical properties of neural tissues. The hydrogels exhibit notably different characteristics compared to nanoparticles reinforced hydrogels. The hydrogels exhibit relatively low modulus, good stretchability, and outstanding fatigue resistance. It is demonstrated that the hydrogels are well suited for fashioning into wearable and implantable sensors that can obtain physiological pressure signals, record the local field potentials in rat brains, and transmit signals through the injured peripheral nerves of rats. The hydrogels exhibit good chemomechanical match to tissues, negligible foreign body response, and minimal signal attenuation over an extended time, and as such is successfully demonstrated for use as long‐term implantable sensory devices. This work facilitates a deeper understanding of biohybrid interfaces, while also advancing the technical design concepts for implantable neural probes that efficiently obtain physiological information.

## Graphical abstract

A strategy is proposed using microgels as large crosslinking centers in hydrogel networks to modulate the tradeoff between modulus and fatigue resistance/stretchability. The prepared hydrogels can be used to obtain physiological signals, record the local field potentials in brains, and transmit signals through injured peripheral nerves. The appropriate performances lead to negligible foreign body response and signal attenuation with time.

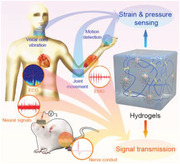

## 1 Introduction

Wearable and implantable sensors are placed to revolutionize health monitoring, disease diagnosis, and precision medicine.^[^ ^1^, ^2^, ^3^, ^4^, ^5^ ^]^ For example, wearable tactile sensors have shown the capability of recording apexcardiogram and sensory monitoring of artery pulse, respiratory rate, joint motion, etc.^[^ ^6^, ^7^ ^]^ Implantable sensors have been used for real‐time in vivo detection of biomarkers, useful for managing chronic diseases, improving personalized drug therapy, and treating neurological disorders.^[^ ^8^, ^9^, ^10^, ^11^ ^]^ For optimized practical performance, wearable and implantable sensors should be fabricated with materials that possess a combination of characteristics, including stretchability, conductivity, fatigue resistance, and biocompatibility. Hitherto, materials that have been investigated to prepare wearable and implantable sensors include metals, metal nanoparticles, conductive polymers, carbon materials, and hydrogels.^[^ ^12^, ^13^, ^14^, ^15^, ^16^ ^]^ The conventional metaland metal nanoparticles sensors have high electronic conductivity, electrochemical stability, and corrosion resistance.^[^ ^17^ ^]^ However, most neural tissues have a modulus of <100 kPa, which is much lower than that of metal electrodes (up to 200 GPa), bulk polymers, and carbon materials.^[^ ^18^ ^]^ For example, when metal electrodes were implanted into brains to record neural signals, the electrodes were encapsulated by glial cells after a certain time causing sensing performance loss.^[^ ^19^ ^]^ The mismatch in chemomechanical properties causes micromotion during long‐term utilization that will damage neural tissues, interfere with sensing results, and trigger adverse biological responses.^[^ ^20^, ^21^ ^]^

Given this challenge, hydrogel‐based electrodes recently attracted much attention, which demonstrates many advantages over the aforementioned insertional counterparts. Hydrogels consist of 3D cross‐linked networks of hydrophilic polymer chains, which can mimic mechanical and chemical properties of brain tissues to reduce micromotion and minimize adverse biological responses.^[^ ^22^ ^]^ As such, hydrogel‐based electrodes have recently been explored to monitor specific frequency ranges of brain neural oscillation.^[^ ^23^ ^]^ The frequency ranges of brain neural oscillation, especially high‐frequency components (>30 Hz), are associated with some cognitive functions and related diseases such as schizophrenia, autism, epilepsy, Alzheimer's disease, etc.^[^ ^24^, ^25^, ^26^, ^27^ ^]^ For example, Anikeeva and coworkers developed hybrid hydrogel probes to long‐term sense and modulate neural activity.^[^ ^28^ ^]^ The probes are comprised of multi‐material fibers coated by a soft poly(vinyl alcohol) hydrogel matrix, which were used to track neuron action potentials and investigate electrophysiological behaviors. The robust hydrogel matrix minimizes brain micromotion and foreign body responses after implantation. Cullen and coworkers prepared implantable “living electrodes” by coating cortical neurons with soft hydrogel cylinders for monitoring/modulating brain activity.^[^ ^29^ ^]^ Bettinger and coworkers prepared polyethylene glycol‐grafted catechol hydrogel‐based multielectrode arrays to record single‐unit neural activity.^[^ ^30^ ^]^ The hydrogel‐based device can conformably contact with neural tissues, thereby improving signal transduction with a minimal autoimmune inflammatory response.^[^ ^31^, ^32^ ^]^

To obtain stable sensing signals, appropriate modulus, good stretchability, and excellent fatigue resistance are the key properties for hydrogel‐based implantable sensors. While foreign body response can be reduced by coating metal or conductive polymers‐based electrodes, they are hard to form compliant conductive interfaces between electrodes and tissues due to higher modulus. The limited interface contact generates an influence on the signal conduction. Highly stretchable hydrogels can form biocompliant hydrogel‐neural tissue interfaces that can reduce the gap between the sensory electrodes and tissues, thus enhancing signal transduction. Although highly stretchable hydrogels can be constructed by supramolecular interactions such as H bonds, coordination bonds, electrostatic interaction, etc., fatigue induced during long‐term applications remains a great challenge.^[^ ^33^, ^34^, ^35^ ^]^ Although some supramolecular hydrogels consisting of cucurbituril host‐guest interactions or quadruple H‐bond crosslinkers can be stretched to 10–100 times longer than their original length, their ability to heal back to their original lengths and morphologies is limited.^[^ ^36^, ^37^ ^]^ The inferior fatigue resistance and deformation of hydrogel‐based electrodes lead to unstable signals and detection variability. Recently, Gong and coworkers demonstrated double‐network hydrogels with high mechanical strength and excellent elasticity.^[^ ^38^, ^39^ ^]^ The hydrogels networks are comprised of amphiphilic polymer chains crosslinked by a combination of electrostatic interaction and covalent bonds. The electrostatic interaction was used as recoverable sacrificial bonds that could reversibly break, re‐form, and dissipate mechanical energy in response to external stretch. The covalent bonds acted as permanent crosslinks allowing for high elasticity and fatigue resistance. In general, the double‐network hydrogels exhibit an elastic modulus of 0.1–1 MPa, which is also larger than that of neural tissues.^[^ ^40^, ^41^, ^42^ ^]^

**Figure 1:**
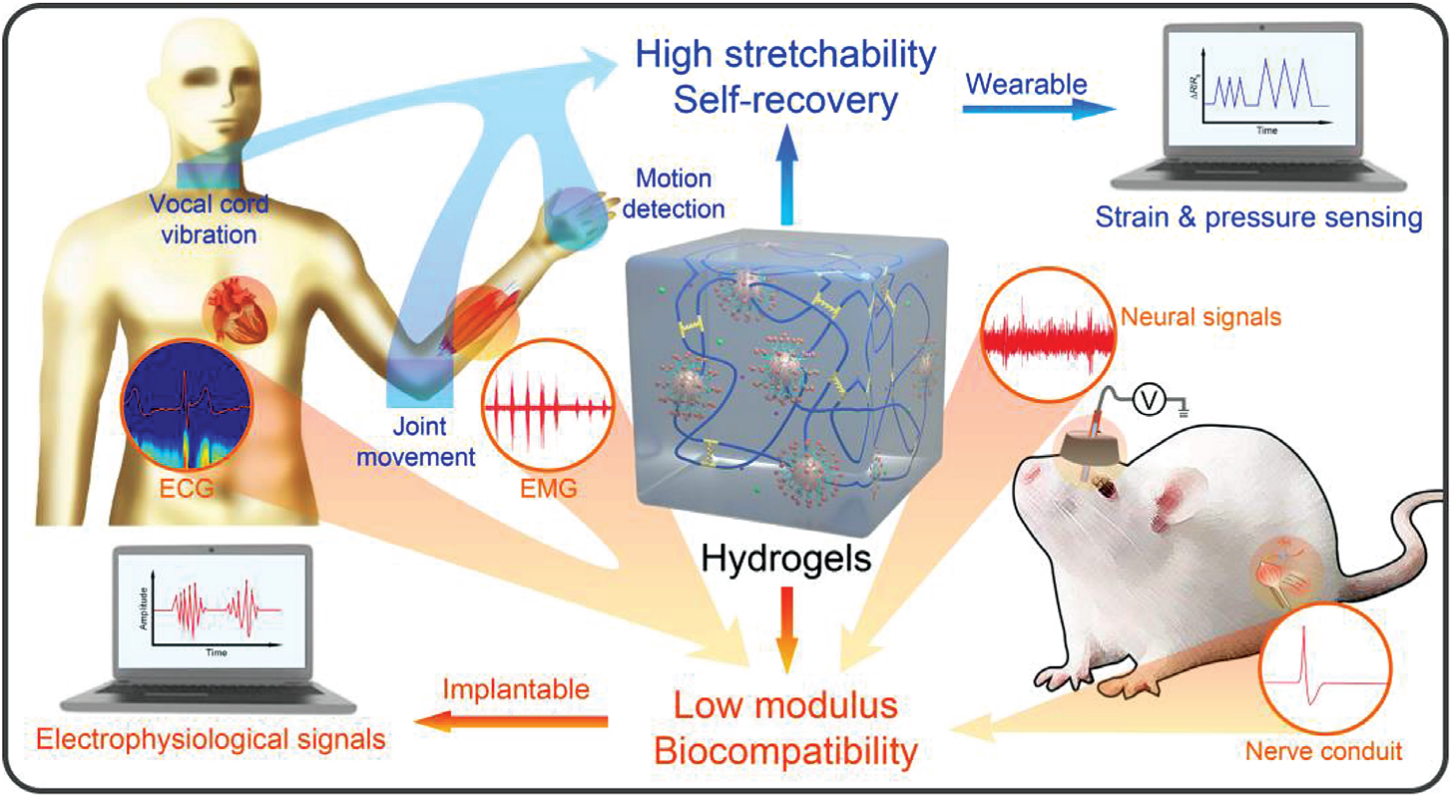
Schematic diagram of the hydrogel structure and its applications as wearable and implantable sensors.

Inspired by the double‐network hydrogels, in this work, we utilize polymer chain entanglements, hydrophobic association, and covalent bonds as crosslinkers to modulate the tradeoff between modulus and fatigue resistance/stretchability. The prepared hydrogels exhibit relatively low modulus, good stretchability, and excellent fatigue resistance, which contain two kinds of crosslinking sites, i.e., microgels (≈600 nm) and lauryl groups. Microgels were used as large crosslinking centers in the hydrogel networks to form polymer chain entanglements and covalent bonds with the hydrogel networks. In comparison to small molecular crosslinkers, the microgels can generate deformation and mechanical buffer when the hydrogels undergo stretch or compression. When the tension is removed, the microgels restore to their original morphology due to their stable crosslinking networks. In previous studies, we have verified that the microgels can change their volume and size in response to mechanical pressure and biomolecules such as uric acid and glucose.^[^ ^6^, ^43^, ^44^, ^45^ ^]^ Capacitive and photonic devices can be fabricated by sandwiching the single‐layer microgel film, which can convert the size changes of microgels into capacitive or optical signals.^[^ ^46^, ^47^ ^]^ The deformation of microgels leads to the high stretchability and excellent fatigue resistance of the hydrogels while maintaining a relatively low modulus. The lauryl groups can form a reversible hydrophobic association that serves as recoverable sacrificial bonds to dissipate mechanical energy, which further increases the hydrogels’ stretchability. We further demonstrate the capability of the hydrogels in the fashioning of wearable and implantable sensors for multipurpose sensing applications, namely (**Figure** **1**): i) The hydrogel‐based wearable sensor could record physiological health signals such as motion, phonation, electrocardiogram (ECG), and electromyogram (EMG). ii) The hydrogel‐based electrodes were implanted into the hippocampus CA1 area of rat brains to record local field potentials (LFPs), which exhibit more stable signals and less immunochemical rejection in comparison to commonly used metallic electrodes during long‐term applications. iii) Nerve guidance conduits (NGCs) were prepared using the hydrogel to bridge two distal stumps of injured sciatic nerves, which exhibit the excellent transmission capability of neural signals.

## 2 Results and Discussions

### 2.1 Preparation and Characterization of Hydrogels

**Figure 2:**
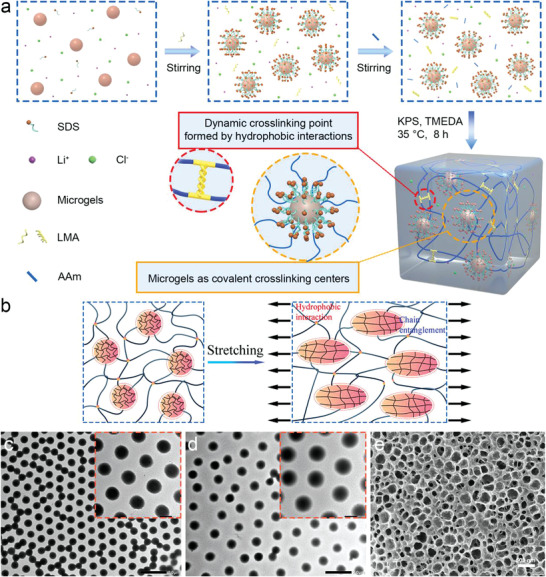
a) The synthesis and structure of hydrogels. b) The schematic deformation of microgels in hydrogel networks by stretching. TEM images of c) microgels and d) vinyl groups modified microgels. e) The SEM image of HM‐2.

The synthesis of the hydrogels and their deformation response to mechanical tension were shown in **Figure** **2**a,b. Initially, the poly(*N*‐isopropyl acrylamide) microgels were synthesized by the precipitation copolymerization of *N*‐isopropyl acrylamide, acrylic acid, and *N*,*N*’‐methylenebisacrylamide. Subsequently, the vinyl groups were attached to the microgel networks by the reaction of carboxyl groups in microgels with 3‐butene‐1‐amine, which was verified by infrared spectroscopy (Figure S1, Supporting Information). The vinyl group content of the microgels was determined to be 0.8 mmol g^−1^ by the nuclear magnetic resonance spectrum (Figure S2, Supporting Information). The size of microgels in the dried state increases from 523 ± 21 to 660 ± 31 nm after the functionalization reaction, as shown in Figure 2c,d. The hydrogels were synthesized by the free radical polymerization of acrylamide (AAm), lauryl methacrylate (LMA), and vinyl groups modified microgels initiated using potassium persulfate (KPS) and tetramethyl ethylenediamine (TMEDA). The hydrogels were noted as HM‐X, where X is the mass percentage of the microgels (Table S1, Supporting Information). The microgels covalently linked with the polymer networks as the vinyl groups participated in the polymerization reaction. The morphology of the synthesized hydrogels was characterized by a cyro‐scanning electron microscope (cyro‐SEM) (Figure 2e). It can be seen that the microgels are uniformly distributed within the hydrogel matrix without any aggregation.

**Figure 3:**
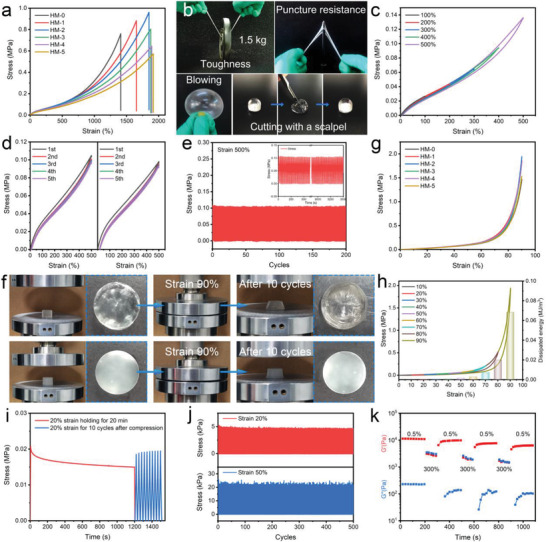
a) Tensile stress‐strain curves of the hydrogels. b) Mechanical performance demonstration of HM‐2 for lifting a weight plate, stabbing with a screwdriver, cutting with a scalpel, and blowing with nitrogen. c) Continuous loading‐unloading tests with strains of 100–500%. d) The fatigue resistance of an original and recovered HM‐2 sample. e) 200 consecutive tensile cycles of HM‐2 under 500% strain. f) Compression photographs of HM‐0 (up) and HM‐2 (bottom). g) Compressive stress‐strain curves of the hydrogels. h) Continuous loading‐unloading tests and calculated dissipated energy at strains of 10–90%. i) Stress relaxation curve at a strain of 20% and ten cycles after 20 min compression. j) 500 consecutive compression cycles of HM‐2 under 20% and 50% strain. k) Cyclic shear strain measurement of HM‐2.

The stress‐strain curves of the hydrogels were recorded to investigate the influence of microgels content on mechanical performance (**Figure** **3**a; Table S2, Supporting Information). In this study, the LMA content was set at 1.0 mol%. The elongation at break increased from 1410% to 1850% with the microgel content increasing from 0 to 2 wt%. An increase in microgel content beyond 2 wt% does not impact elongation. The tensile strength of the hydrogels first increases and then decreases with an increase in the microgel content from 0% to 5%. The maximum tensile strength was obtained for HM‐2 samples. HM‐2 exhibits an elastic modulus of 59.7 kPa, a tensile strength of 1.01 MPa, and an elongation at break of 1866%. The elastic modulus of all hydrogel samples was determined to be in the range of 47.0–59.7 kPa, which matches the elastic modulus of biological neural tissues (1–100 kPa). The mechanical performances of HM‐2 and other recently reported wearable or implantable hydrogels have been shown in Figure S3 in the Supporting Information.^[^ ^48^, ^49^, ^50^, ^51^, ^52^, ^53^, ^54^, ^55^ ^]^ It can be seen that the microgels exhibit different influences on mechanical performance in comparison to traditional rigid nanofillers such as carbon nanotubes, MXene, cellulose nanocrystals, etc. The microgels lead to a larger stretchability and retain a relatively lower elastic

modulus in comparison to traditional rigid nanofillers. This phenomenon was attributed to the deformation capability of soft microgels as macro‐crosslinkers in response to external stress.^[^ ^56^, ^57^, ^58^ ^]^ The covalent cross‐linkage and chain entanglement of the microgels serve as a self‐recovery component. When the tension was removed, the microgels rapidly restore to their original morphology. The robust mechanical performance of HM‐2 has been shown in Figure 3b. A 1.5 kg barbell plate was lifted using a HM‐2 sheet of 5.0 cm × 1.0 cm × 0.2 cm, which attests to the hydrogel material's superior toughness. HM‐2 also exhibits strong puncture resistance. The HM‐2 sheet withstood vigorous puncturing by a screwdriver with no observable damage. The HM‐2 sheet deformed and inflated into a balloon when exposed to a N_2_ flow. In addition, HM‐2 could be squeezed by sharp scalpel blades without leaving marks, as an evidence to its excellent scratch resistance.

Subsequently, a suit of tensile loading‐unloading tests was carried out, as shown in Figure 3c. As the maximum strain increases from 100% to 500%, evident hysteresis loops were found which indicates effective energy dissipation by the destruction and reconstruction of hydrophobic association and microgels deformation. The hysteresis loops nearly overlap for the five tensile test cycles, which evidence the HM‐2 ability to maintain the same interior networks structure during the test. After 5 min rest, the HM‐2 sheet was subjected to another five continuous loading‐unloading tests at a strain of 500%, exhibiting similar hysteresis loops to the first five cycles test (Figure 3d). The synergistic effect of hydrophobic association and microgels deformation generates the excellent self‐recovery properties of HM‐2. In addition, the fatigue resistance of HM‐2 was evaluated by conducting 200 consecutive tensile testing cycles at the strain of 500% (Figure 3e) and 500 consecutive tensile testing cycles at the strain of 100% (Figure S4, Supporting Information). No obvious performance loss was observed for these tests, which indicates outstanding fatigue resistance and self‐recovery capability. Subsequently, consecutive compression tests were performed on HM‐2 to examine its elasticity, self‐recovery, and fatigue resistance. The irreversible deformation of HM‐0 (samples without microgels) was found after approximately ten compression cycles with 90% strain, and obvious cracks and damage were observable on the hydrogel surface (Figure 3f). Meanwhile, HM‐2 could restore to its original morphology with no observable damage following the same magnitude of compression. Then HM‐2 were subjected to cyclic compression tests with different strains. The compressive stress‐strain curves are shown in Figure 3g. The compression stress and hysteresis loop rise with increased strain, which exhibits a typical elastic characteristic (Figure 3h). The compression stress and dissipated energy at 90% strain were up to 1.94 MPa and 0.068 MJ m^−3^, respectively. To further investigate the capacity for self‐recovery after compression, the HM‐2 was first compressed at 20% strain and held for 20 min, and then immediately subjected to ten compressive loading‐unloading cycles. As shown in Figure 3i, the stress relaxation occurs under long‐term external force, which stems from the break and reconstruction of the dynamic cross‐linked network.^[^ ^59^ ^]^ After 20 min compression, HM‐2 showed the same elastic behavior as the original state. 500 continuous compression tests at the strains of 20% and 50% were conducted, respectively, as shown in Figure 3j. HM‐2 exhibits a constant compressive strength during the loading‐unloading process. These results demonstrate the excellent fatigue resistance of HM‐2 against tension and compression, which allow the hydrogel as a suitable material to obtain stable signals with minimal baseline drifts during long‐term sensory applications.

Rheological tests were conducted on the hydrogels to further investigate viscoelastic properties, fatigue resistance, and self‐recovery performance at 25°C. The viscoelastic properties of the hydrogels were evaluated by oscillation angular frequency sweep tests (Figure S5, Supporting Information). It can be found that the storage modulus (*G*′) was insensitive to frequency changes and was always larger than the loss modulus (*G*′′) over the entire frequency range (0.1–100 rad s^−1^). This result indicates the elastic response as the predominant performance for these hydrogels.^[^ ^60^, ^61^ ^]^ The *G*′ and *G*′′ were determined to be in the range of 8.4–13.9 kPa and 430–840 Pa, respectively. HM‐2 exhibited the largest *G*′, which is consistent with the tensile and compression results. Strain amplitude sweep tests were performed on HM‐2 in the strain of 0.1–1000% to investigate the limit of the linear viscoelastic region (Figure S6, Supporting Information). The *G*′ and *G*′′ values of HM‐2 were constant as the shear strain increased from 0.1% to 30%, which suggests HM‐2 in a gel state. The intersection points of *G*′ and *G*′′ curves were observed at the strain of 98% for HM‐0 and 170% for HM‐2, which correspond to the critical points between the gel and sol states.^[^ ^62^ ^]^ These results also verified that the microgels play an important role in enhancing the stretchability and flexibility of the hydrogels. In addition, cyclic shear strain testing was applied to investigate the self‐recovery performance (Figure 3k). As shown in Figure 3k, a small strain of 0.5% for 200 s and a large strain of 300% for 100 s were alternatively applied to HM‐2. HM‐2 was in a sol and gel state at large shear strain and small shear strain, respectively. After switching from 300% strain to 0.5% strain, the modulus recovered to its original value within 30 s. Due to its excellent mechanical performance, HM‐2 was further investigated for sensory applications.

### 2.2 Sensing Performance as Tactile and Wearable Sensors

**Figure 4:**
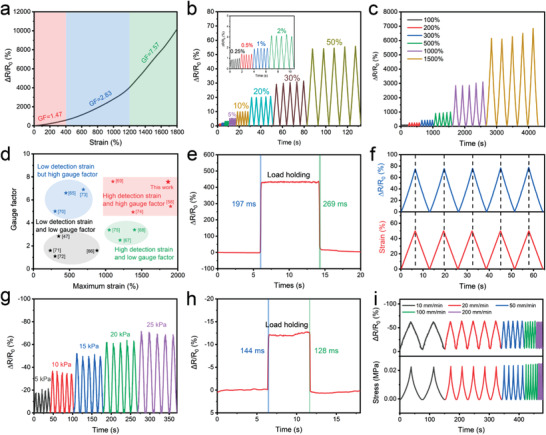
a) Resistance changes as a function of strains for HM‐2. b) The resistance changes of HM‐2 at strains of 0.25–50%. c) The resistance changes of HM‐2 at the strain of 100–1500%. d) Comparison of HM‐2 sensor with other reported hydrogel strain sensors in terms of maximal strain and gauge factor.^[^ ^49^, ^59^, ^66^, ^67^, ^68^, ^69^, ^70^, ^71^, ^72^, ^73^, ^74^, ^75^, ^76^ ^]^ e) Response time and recovery time of the HM‐2 strain sensor. f) Electrical hysteresis of HM‐2 at a strain of 50%. g) Five compressing and releasing cycle tests of HM‐2 in the pressure range of 5–25 kPa. h) Response time and recovery time of the HM‐2 pressure sensor. i) The resistance and stress changes of HM‐2 at a suite of compression speed under a pressure of 20 kPa.

To realize the sensing applications, inorganic ions (e.g., acids, bases, metal ions, etc.) have been used as electrolytes in hydrogels to achieve high ion conductivity.^[^ ^48^, ^63^, ^64^ ^]^ It has been reported that artificial cerebrospinal fluid (ACSF) is a biocompatible electrolyte used in recording extracellular electrophysiological signals.^[^ ^65^ ^]^ We have here demonstrated the use of ACSF electrolytes within HM‐2 to achieve high ionic conductivity. A HM‐2 sheet infused with ACSF was used as a conductor to connect with a LED, as shown in Figure S7 and Videos S1 and S2 in the Supporting Information. When the HM‐2 sheet was stretched or compressed, the brightness of the LED light significantly changed. This phenomenon stems from the resistance changes of the HM‐2 sheet caused by its deformation. The relationship between resistance change (Δ*R*/*R*_0_) and tensile strain has been shown in **Figure** **4**a. The curve can be roughly divided into three linear regions, and the corresponding gauge factors (GF) are 1.47 (0–400%), 2.83 (400%‐1200%), and 7.57 (1200%‐1800%). The linear change of Δ*R*/*R*_0_ in the specified strain intervals enables the HM‐2 sheet to adapt to the deformation of the epidermis and tissues, which is an important characteristic for wearable devices applications. The repeatability of the HM‐2 sensor was evaluated in the strain range of 0.25%‐1500% (Figure 4b,c). The HM‐2 sensor was stretched five times at each strain and similar curves of resistance changes were observed suggesting excellent repeatability in the whole strain range. High GF value and wide detection range are two important strain sensor performance criteria. Herein, the performances of the HM‐2 sensor and other recently reported strain sensors were compared, as shown in Figure 4d. The HM‐2 sensor shows a larger GF value and wider detection range compared to other strain sensors. It should be noted that the HM‐2 sensor demonstrates fast response (197 ms) and short recovery time (269 ms) and does not exhibit electrical hysteresis during the tensile test, which are desirable characteristics as strain sensors (Figure 4e,f). To investigate durability, 500 continuous tensile testing cycles at the strain of 100% (Figure S8, Supporting Information) were conducted. No performance loss was observed during the test, which indicates excellent durability and stability. The pressure sensitivity of the HM‐2 sensor has also been investigated and the results are shown in Figure S9 in the Supporting Information. The HM‐2 sensor exhibits the sensitivity of 0.17 and 0.03 kPa^–1^ in the ranges of 0–2 and 2–10 kPa, respectively. For the compression test, the HM‐2 sensor shows a good reproducibility of pressure response with fast response (144 ms) and short recovery time (128 ms) (Figure 4g,h). Moreover, as the tensile or compression rate increases from 10 to 200 mm min^−1^, the Δ*R*/*R*_0_ of the sensor remains almost unchanged (Figure 4i). These results indicate that the Δ*R*/*R*_0_ is independent of tensile or compression rate. Following 500 continuous compression testing cycles at the strains of 20% and 50%, the HM‐2 sensor maintained an excellent reproducibility after 30 min testing (Figures S10 and S11, Supporting Information). All aforementioned results indicate the excellent repeatability and stability of the HM‐2 as tactile sensors.

**Figure 5:**
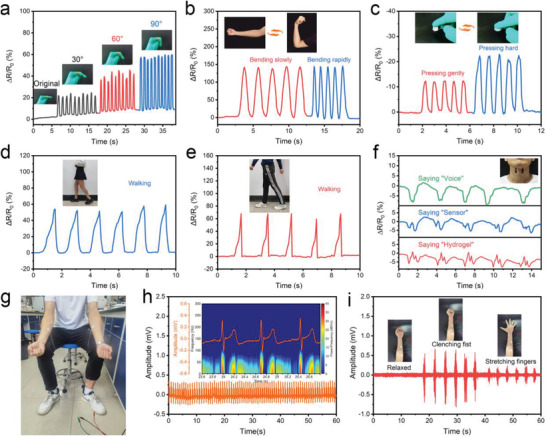
a) Changes in HM‐2 resistance in response to finger bending at the angles of 30°, 60°, and 90°. b) Change in HM‐2 resistance in response to elbow bending and releasing. c) Change in resistance when a finger presses the HM‐2 sensor. The real‐time resistance signals when the wearers were d,e) walking and f) speaking. g,h) The ECG detection and results. i) The EMG signals of a volunteer for conducting some gestures.

The HM‐2 superior mechanical performance lends it for use as a wearable physiological signal device. As shown in **Figure** **5**a, when an HM‐2 sheet was attached to an index finger joint, its resistance increased rapidly as the bending angle changed from 0° to 90°. Similarly, HM‐2 showed the capability of monitoring elbow movements (Figure 5b). The bending angles and frequency (or rate) could be obtained from the resistance signals that result from the tensile and compression deformation of the HM‐2 sheet. The excellent compression fatigue resistance allows HM‐2 to be used for monitoring body movements such as touching and walking (Figure 5c–e). As shown in Figure 5d,e, when an HM‐2 sensor was attached to the heels of a female (50 kg) and a male (75 kg) undergoing a walking exercise, characteristic peaks were obtained. Following each walking step, the characteristic peak shapes and intensity correlate with the subjects' distinct gestural motion and body weight, respectively. As such, the HM‐2 sensor's walking spectrum can provide information about walking frequency, body weight, and walking gestural motion. The HM‐2 sensor was further used to monitor the vibration of the vocal cords and subtle epidermis deformation during speaking for sound recognition, of importance in vocal cord recovery and televox applications. When the HM‐2 sensor was attached to a volunteer's throat, a suite of characteristic peaks was obtained on the phonetics of specific words (Figure 5f). Each word was repeatedly spoken five times and the same peak shape was found for each word indicating good repeatability. These words can be clearly distinguished by the resistance signal spectrum demonstrating the capability of the HM‐2 sensor for sound recognition. Due to the high conductivity and stretchability, HM‐2 was made into electrode pads with a diameter of 1.5 cm and a thickness of 2 mm to record electrophysiological signals such as ECG and EMG. Three HM‐2 electrode pads were attached to the left/right arms and left ankle of a 27‐year‐old male to record the ECG, as shown in Figure 5g. Typical ECG waveforms were obtained using the HM‐2 electrode pads including P‐wave (atrial depolarization), QRS complex (ventricular depolarization), and T‐wave (ventricular repolarization) (Figure 5h). The clear frequency identification of P‐QRS‐T peaks is achieved in the power density range of 20–40 dB Hz^−1^, a very important indicator in diagnostics of cardiac diseases like myocarditis and arrhythmia. The HM‐2 electrode pad was further used for EMG to monitor the action potential produced by muscle fibers. Three HM‐2 electrode pads were attached to the flexor muscle (left arm) of a volunteer. The volunteer was directed to clench and release his fist with an interval of 3 s for 1 s (Figure 5i). Three dominant EMG bursts were found in the EMG. The process of fist‐clenching and releasing leads to obvious changes in potential intensity. The signal‐noise ratio of EMG (SNR_EMG_) recorded by the HM‐2 electrode pads was calculated based on the ratio of the signal amplitude to the baseline noise. The SNR_EMG_ of 18.1 dB was obtained when clenching fists, which even exceeds that recorded by commercial Ag/AgCl electrode (13.1 dB).^[^ ^77^ ^]^ These results show that the HM‐2 electrode pads can collect high‐quality electrophysiological signals.

### 2.3 Sensing Performance as Implantable Brain Neural Sensors

**Figure 6:**
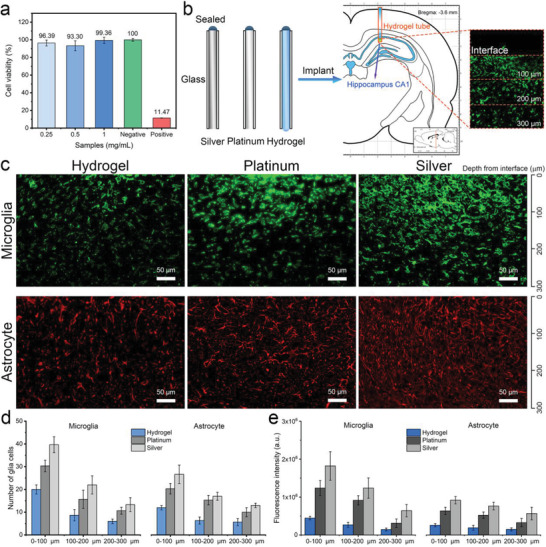
a) The viability of RSC96 cells in a suite of leaching solutions for 24 h. b) Schematic diagram of the implantation position of these electrodes for the biocompatibility investigation. c) Confocal micrographs of the brain slices: microglia cells (green) and astrocyte cells (red). d) The statistical numbers of microglia and astrocyte cells within the square area of 300 × 300 µm in the depth range of 0–100, 100–200, 200–300 µm from these electrodes. e) The mean fluorescence intensity of the confocal images corresponding to the cell counting area.

To further investigate the capability of implantable sensors, we evaluated the in vitro cytotoxicity of HM‐2 by measuring the viability of RSC96 cells (**Figure** **6**a; Figure S12, Supporting Information). The cells were cultured in a suite of leaching solutions for 24 h, after which the cell morphology and proliferation were studied. The live and dead (L/D) assay demonstrates only viable cells (green stained) and almost no dead cells (red‐stained) in the experimental groups. The cell viability is larger than 93% after 24 h culture in all experiments, attesting that HM‐2 has negligible cytotoxicity to RSC96 cells. We further investigated the immune response of the HM‐2, silver, and platinum electrodes when implanted in the rats' hippocampus CA1 area (Figure 6b). After 1 week, the hippocampus tissues that contacted the electrodes were sliced, immunochemically treated, and investigated using a confocal microscope. As shown in Figure 6c, a less immunochemical reaction was observed for platinum electrodes compared to the silver counterparts, which is consistent with the literature.^[^ ^78^ ^]^ The HM‐2 electrode showed much less

immunochemical reaction than platinum. In the brain tissue, glial cells (microglia and astrocytes) tend to migrate to a foreign body as a physiological immune response phenomenon.^[^ ^79^, ^80^ ^]^ The numbers of glial cells within the square areas of 300 × 300 µm were determined in the depth range of 0–100, 100–200, 200–300 µm relative to the position of the electrode. As shown in Figure 6d, the numbers of glial cells near HM‐2 electrodes are significantly less than those near silver and platinum electrodes. Meanwhile, the slices contacting HM‐2 show the weakest mean fluorescence intensity compared to those contacting platinum and silver slides (Figure 6e), which evidences the negligible cytotoxicity and excellent biocompatibility of the HM‐2.

**Figure 7:**
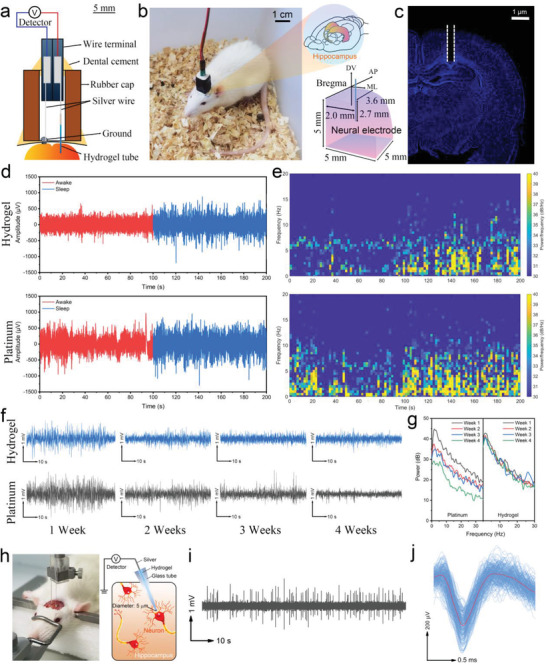
Detecting neural signals of rats in vivo. a) Schematic diagram of the device for detecting the LFPs of free‐moving rats. b) Photograph of a rat implanted with an HM‐2 electrode and the indication of the implant position. c) A micrograph of the rat brain implanted with an HM‐2 electrode, using DAPI as a nuclear fluorescent stain. d) LFPs recorded by HM‐2 and platinum electrodes. e) The spectrograms of the signals in Figure 7d. f) The LFP signals recorded in the sleep state by HM‐2 and platinum electrodes for 4 weeks of detection. g) The power corresponding to (f). h) The device for recording neuronal spikes in vivo. i) The neuronal spikes of a rat recorded in vivo. j) The result of spike sorting: overlay spike signals (blue) and the average waveform of the sorted spikes.

The excellent biocompatibility and less immunological rejection lend the HM‐2 electrode as a suitable neural probe for recording LFPs in rats' brains. LFPs are produced by transient imbalances in extracellular ion concentrations due to cellular electrical activity. Ion exchange between HM‐2 electrodes and neuron cells can occur when the neuron cellular action potential generation. The action potential can be converted into electric signals by HM‐2 electrodes via ion diffusion. To achieve stable neural signals, a designed setup comprising a HM‐2 electrode and a reference electrode was fabricated and inserted in the hippocampus CA1 area of rats for long‐term LFPs monitoring (**Figure** **7**a,b). LFPs are used to index the comprehensive electrophysiological activity of local brain regions including massive neurons within a few hundred microns to a few millimeters near the electrodes.^[^ ^81^ ^]^ Consequently, the HM‐2 electrode was prepared in a flexible fused silica capillary with the inner and external diameters of 320 and 450 µm to contact sufficient neurons for obtaining LFPs. The flexible and insulating polyimide coated silica capillary can shield signal interference from other areas, ensuring pick up of signal response obtained through the electrode tip. The HM‐2 fused silica capillary‐based electrode was implanted in the hippocampus CA1 regions of free‐moving rats (Figure 7c). Clear LFPs signals were obtained by the HM‐2 electrode, with noticeable differences in the wake and sleeping states of the rats (Figure 7d,e; Videos S3 and S4, Supporting Information). The LFPs show lower frequencies (2–7 Hz) and larger amplitudes in the sleeping state, associated with the simultaneous activity of a large number of neurons in the sleeping state. It has been reported that animals produce theta rhythm in LFP signals during sleeping.^[^ ^82^, ^83^ ^]^ For comparison, LFPs signals were also recorded using a platinum electrode, as shown in Figure 7d. The platinum electrode also captured similar LFP signals with that obtained by the HM‐2 electrode. However, the platinum electrodes resulted in large LFP signal drifts due to chemomechanical interfacial mismatch between the brain tissue and the metal electrode. Evidently, compared to platinum, the HM‐2 electrode exhibits excellent stable LFP signals for rats in motion states (Video S5, Supporting Information). To investigate long‐term performance, the HM‐2 and platinum electrodes were implanted into the hippocampus CA1 regions of rat brains and the LFP signals were recorded for 4 weeks. The changes in LFP signals in a sleep state with time have been shown in Figure 7f. Although platinum electrodes produced LFP signals with higher intensity for the first two weeks, their intensity decreased significantly over time to much lower levels compared to HM‐2 electrodes (Figure 7g). The HM‐2 electrode on the other hand yielded relatively stable LFP signals for the 4 weeks. This confirms that the HM‐2 electrode is suited as an implantable sensor for long‐term brain neural sensory applications. After the 4 weeks of implantable detection, the hippocampus tissues were sliced to investigate immune response and illustrate the reason for LFP signal attenuation over time. As shown in Figure S13 in the Supporting Information, the number of glial cells observed at the HM‐2 electrode interface was significantly less than those determined for platinum. The less glial cell coverage leads to stable LFP signals obtained by HM‐2 electrode over the 4 weeks of implantable detection.

Besides LFPs, the neuronal spike is another important brain neural signal that is assumed to be the main inter‐neuronal communication pathway.^[^ ^84^, ^85^ ^]^ Neuronal spikes detection from a single neuron is essential in decoding and understanding cognition and brain neuron communication pathways. HM‐2 microelectrode was investigated to record spikes of a single neuron in the hippocampus CA1 regions (Figure 7h). The size of a rat's hippocampal neuron ranges from ≈20 µm (cell body) to more than 150 µm (dendrites).^[^ ^86^ ^]^ To match the microsampling single neuron region, a microelectrode was prepared by filling HM‐2 in a glass capillary with a diameter of 5 µm. When the tip of the HM‐2 microelectrode was close enough to a neuron, clear spikes were observed (Figure 7i). The recorded potential signals were sorted using an algorithm published by Toosi et al.^[^ ^87^ ^]^ To obtain a statistical waveform neural spikes waveform that could be characterized, ≈210 spikes were overlapped (Figure 7j). The obtained spikes waveform was consistent with that of neuronal firing reported by Zátonyi et al.^[^ ^88^ ^]^ The signal‐to‐noise ratio of spike signals (SNR_spike_) is defined as the ratio of the effective power of neuronal spikes to that of noise. The SNR_spike_ of the HM‐2 microelectrode was determined to be 7.2 dB, which closely matches what has been reported for microelectrodes made from gold, platinum, graphene, etc.^[^ ^88^, ^89^, ^90^ ^]^ As such, the HM‐2 electrodes demonstrated unique capability for probing LFPs and single neuronal spikes.

### 2.4 Transmission Performance of Neural Signals through Injured Peripheral Nerves

**Figure 8:**
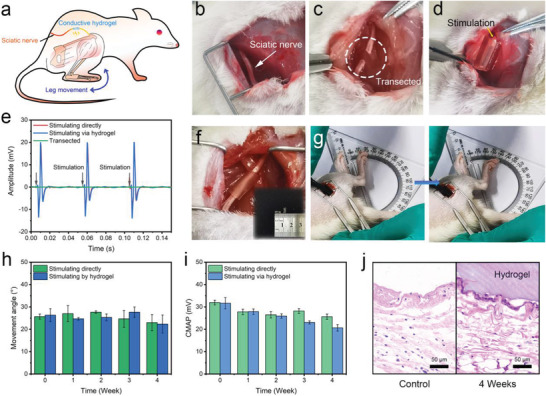
The neural signal conduction ability and the performance stability of HM‐2 for long‐term implantation. a) Schematic illustration of the sciatic nerve stimulation setup. b) The intact sciatic nerve. c) A sciatic nerve defect model with a gap defect of 1 cm in the right leg. d) Two distal stumps connected by an NGC. e) CMAP signals while stimulating the sciatic nerve with a monophasic electrical waveform (5 ms single pulse). f) Image of a rat sciatic nerve wrapped with an NGC. g) Images of the ankle joint movement in response to electrical stimulation via the HM‐2 sheet. h) The response of movement angle to electrical stimulation for different implantation time. i) The response of CMAP peak signal to electrical stimulation for different implantation time. j) H&E histological results of the control and HM‐2 samples after 4 weeks of implantation, respectively.

Another important application was also explored to utilize HM‐2 for the transmission of neural signals through injured peripheral nerve tissues. The peripheral nerve is incredibly important in transmitting information from the brain to the rest of the body. Peripheral nerve injuries are known to cause neurological disorders, chronic pain, paralysis, and even disability.^[^ ^91^ ^]^ In this study, a sciatic nerve flaw was created by introducing a gap defect of 1 cm in the rat's right legs. NGCs were prepared using HM‐2 to connect the two distal stumps of the sciatic nerve gap defect (**Figure** **8**a–d). The electrophysiologic assessment was conducted to investigate the transmission of neural signals, which was probed by measuring the compound motor action potential (CMAP). When the normal sciatic nerve was stimulated, a CMAP of ≈30 mV was obtained, as shown in Figure 8e. When the sciatic nerve was transected, no CMAP signal was observed due to the transmission disruption. After applying the NGCs, the same CMAPs and response time as normal nerves were achieved, which indicates the excellent performance of neural signal transmission through the injured peripheral nerves. To further investigate the signal transmission capability for long‐term application, a HM‐2 sheet was tightly wrapped on an intact sciatic nerve and the response of CMAP and leg movements to electrical stimulation was recorded with time (Figure 8f,g). The intact sciatic nerve, rather than the injured sciatic nerve, was chosen for this study to avoid the interference of nerve cell growth with time. When a 5 voltage was applied to the HM‐2 sheet, a gastrocnemius contraction was induced by the action potential (Figure 8g; Videos S6 and S7, Supporting Information). A similar bending angle (≈25°) of the right legs was observed when the electrical stimulation was applied on the HM‐2 sheet and sciatic nerve, respectively. No decrease in bending angle was found for 4 weeks of implantable tests (Figure 8h), which indicates the signal transmission capability for long‐term applications. In the third week of the electrical stimulation experiment, a slight decrease in CMAPs was noted in the sciatic nerve wrapped by the HM‐2 sheet (Figure 8i). The phenomenon stems from the inevitable cell adhesion and proliferation on the hydrogel surface, which increases the electrode interface impedance. However, there was no observable inflammatory response during the four weeks of HM‐2 implantation (Figure 8j), which further demonstrates the excellent neural signal transmission capability for long‐term applications.

## 3 Conclusion

A strategy was developed by using microgels as large crosslinking centers in hydrogel networks which have advanced biomimetic chemomechanical performance compared to hydrogels reinforced by inorganic fillers. The hydrogels display relatively low modulus but outstanding stretchability and excellent fatigue resistance. These enhanced performance indicators lend the hydrogel materials to be suitable for wearable and implantable applications. The hydrogels exhibit the ability to detect various physiological information such as phonation, electrocardiograph, and electromyography, which are useful personalized wellbeing diagnostics indicators. The close match in chemomechanical properties between the hydrogel electrodes and brain neural tissues reduces the micromotion, enhances the signal transmission through biohybrid interfaces, and minimizes foreign body response, thereby making the hydrogels especially suitable for implantable applications. The hydrogel electrode implanted in the rat's brain hippocampus CA1 regions was successfully demonstrated for in situ recording LFPs for 4 weeks while yielding stable signals and lower signal attenuation with time. The hydrogel electrodes exhibit excellent neural signals transmission performance. When the hydrogel‐based NGCs were used to bridge two distal stumps of an injured sciatic nerve, CMAP data that was comparable to an intact sciatic nerve was obtained, with no observable signal transmission loss during the 4 weeks of implantation. All of the successful applications stem from the intrinsic characteristics of the advanced hydrogels such as the well‐matched mechanical‐chemical properties with neural tissues, excellent stretchability, and outstanding fatigue resistance. The advances demonstrated in this work will conduce to understand the signal transmission through biohybrid interfaces and induce more opportunities for the studies of human‐machine interaction.

## Conflict of Interest

The authors declare no conflict of interest.

## Supporting information

Supporting Information

Supplemental Video 1

Supplemental Video 2

Supplemental Video 3

Supplemental Video 4

Supplemental Video 5

Supplemental Video 6

Supplemental Video 7

## Data Availability

The data that support the findings of this study are available from the corresponding author upon reasonable request.
