# Supplementary material for: Highly Stretchable Hydrogels as Wearable and Implantable Sensors for Recording Physiological and Brain Neural Signals
Source: Adv Sci (Weinh). 2022 Mar 31;9(16):2201059. doi: 10.1002/advs.202201059 (PMC9165511; doi:10.1002/advs.202201059)
Supplement: Supplementary file 1 — Supporting Information [file ADVS-9-2201059-s005.pdf]

Supporting Information

Highly Stretchable Hydrogels as Wearable and Implantable Sensors for Recording Physiological and Brain Neural Signals

Quanduo Liang, Xiangjiao Xia, Xiguang Sun, Dehai Yu, Xinrui Huang, Guanghong Han, Samuel M. Mugo, Wei Chen, and Qiang Zhang**

Materials

N-Isopropylacrylamide (NIPAm), *N*,*N*'-methylenebisacrylamide (MBA), acrylic acid (AAc), potassium persulfate (KPS), 1-ethyl-3-(3-dimethylaminopropyl) carbodiimide hydrochloride (EDC), N-hydroxysuccinimide (NHS), 3-butenylamine hydrochloride, calcium chloride dihydrate ($CaCl_2 \cdot 2H_2O$), sodium bicarbonate ($NaHCO_3$), sodium dodecyl sulfate (SDS), acrylamide (AAm), and *N*,*N*,*N*',*N*'-tetramethyl ethylenediamine (TMEDA) were purchased from Aladdin. Trimethylamine (TEA) was purchased from Macklin. Lauryl methacrylate (LMA) was purchased from Acros. Sodium chloride (NaCl), potassium chloride (KCl), magnesium chloride hexahydrate ($MgCl_2 \cdot 6H_2O$), sodium dihydrogen phosphate (NaH_2PO_4), and glucose monohydrate (glucose·H_2O) were purchased from Sinopharm Chemical Reagent Co., Ltd. Fixative solution (4% formaldehyde, BL539A) was purchased from Biosharp. All reagents were used as received.

Synthesis of microgels

The microgels were synthesized according to a previous procedure with minor modification.[1] NIPAm (24 mmol, 2.712 g), AAc (2.4 mmol 170 μL), and MBA (1.44 mmol, 222 mg) were dissolved in 198 mL deionized water and then filtered through a 0.2 μm filter. The solution was bubbled with N_2 to remove dissolved O_2 in the solution. The solution was heated to 70 °C and KPS (0.4 mmol) in 2 mL deionized water was added to initiate the polymerization reaction. The reaction ensued for 3 h at 70 °C in an N_2 atmosphere. The

resulting suspension was cooled to room temperature and then filtered to remove aggregated particles. The microgel suspension was purified by centrifugation to remove unreacted monomers. The cleaned microgels were stored in a brown glass jar for future use.

Synthesis of vinyl groups modified microgels

50 mL microgel suspension (1.2 mmol -COOH) was bubbled by N_2 for 30 min to remove O_2 from the system. EDC (19.2 mmol, 3.68 g) and NHS (4.8 mmol, 552 mg) were added to activate carboxyl groups. After stirring for 2 h, 3-butenylamine hydrochloride (2.4 mmol, 258 mg) was added, and then the pH of the suspension was adjusted to ~8 by TEA. The reaction proceeded at room temperature for 8 h in an N_2 atmosphere. The microgel suspension was purified by centrifugation to remove unreacted monomers and catalysts. The cleaned microgels were stored in a refrigerator for future use.

Preparation of artificial cerebrospinal fluid (ACSF):

The ASCF used in our experiments was prepared according to the methodology described by Sheng et al.[2] The ASCF was prepared in deionized water containing (in mM): 125 NaCl, 2.5 KCl, 2 $CaCl_2 \cdot 2H_2O$ 1.3 $MgCl_2 \cdot 6H_2O$ 1.3 NaH_2PO_4, 25 $NaHCO_3$, and 10 glucose·H_2O. The solution was allowed to stand for 24 h and the supernatant was taken for use. The ACSF had a pH value of 7.45.

Synthesis of hydrogels

The hydrogels were synthesized by a micellar-copolymerization method. Briefly, SDS (0.58 g, 2.0 mmol) was dissolved in 30 mL ACSF, and the mixture was stirred for 20 min. Then, LMA (1 mmol, 290 μL) and microgel suspension (0-7.5 g) were added to the SDS/ACSF solution. The mixture was sonicated for 30 min and stirred at room temperature for 3 h to make the microgels evenly dispersed. Subsequently, AAm (7.11 g, 0.10 mol) was

added and the mixture was stirred for 10 min, and then KPS (27 mg, 0.10 mmol) and TMEDA (15 μL, 0.10 mmol) were further added with stirring for 5 min. The obtained mixture was injected into the mold consisting of two parallel glass plates and a 2 mm silicone spacer and then polymerized at 35 °C for 8 h. The compositions of hydrogels are listed in **Table S1**.

Table S1. The compositions of the hydrogels.

Samples	ACSF [mL]	SDS [g]	Microgels [g]	LMA [μL]	AAm [g]	KPS [mg]	TMEDA [μL]
HM-0	30	0.58	0	290	7.11	27	15
HM-1	30	0.58	1.5	290	7.11	27	15
HM-2	30	0.58	3.0	290	7.11	27	15
HM-3	30	0.58	4.5	290	7.11	27	15
HM-4	30	0.58	6.0	290	7.11	27	15
HM-5	30	0.58	7.5	290	7.11	27	15

Characterizations

The Fourier transform infrared (FT-IR) spectra of microgels and vinyl groups modified microgels were obtained using a Nicolet 5700 spectrometer set at 4000-600 cm^{-1} with a resolution of 0.4 cm^{-1}. Nuclear magnetic resonance (NMR) spectra were recorded at room temperature using a Bruker Avance II spectrometer operating at a frequency of 500 MHz. The dispersions of microgels were concentrated and lyophilized into powders before the test. Transmission electron microscope (TEM) analysis was performed on a JOEL JEM1400. The morphologies of the hydrogels were performed using a ZEISS GeminiSEM. The mechanical properties of the hydrogels were evaluated using a universal testing machine (Instron 5982). Hydrogel specimens with a width of 2 mm were prepared for the tensile test at a crosshead rate of 100 mm/min and a standard length of ~20 mm. The values presented are the arithmetic means (± standard deviation) from at least three replicates of every sample. The compression test was carried out with the same instrument. The samples were prepared into a cylinder with a diameter of ~20 mm and a height of ~10 mm. The compression speed was set to 10 mm/min. The resistance of the hydrogel sensors was characterized by combining the universal testing machine and an LCR digital bridge (TH2830, Tonghui Electronics Co., Ltd.). The sensitivity of the hydrogel strain sensor is characterized by the gauge factor (GF). The calculation formula of the GF is:

$$\mathrm{GF} = \frac{\Delta R/R_0}{\varepsilon} \quad (1)$$

where ΔR is the resistance change, R_0 is the original resistance, and ε is the strain of the sensor. The ionic conductivity of the hydrogels was detected by an electrochemical workstation (CH Instruments) using AC impedance mode. The average value of the impedance at a scanning frequency range of 10^4-10^5 Hz was regarded as the resistance of the test sample. The electrocardiogram (ECG) and electromyogram (EMG) signals were obtained by an

RM6240EC multi-channel physiological signal acquisition system (Chengdu Instrument Factory), with signals acquired at an acquisition frequency of 8 kHz and 20 kHz, respectively.

Preparation of the hydrogel electrodes

The precursor solution of HM-2 was injected into pulled glass capillaries (Sutter) with a tip diameter of ~5 μm to prepare HM-2 microelectrodes for detecting neuronal spike. In addition, fused silica capillaries (320 μm in inner diameter and 450 μm in outer diameter) were immersed in the precursor solution of HM-2 to prepare HM-2 electrodes for detecting the local field potentials (LFPs). After polymerization at 35 °C for 8 h, all the prepared electrodes were immersed in deionized water for 7 days, and the water was changed every 12 h to remove unreacted monomers, after which the electrodes were stored in ACSF before use.

Ethical statements

The wearable tests were approved by the Institutional Ethics Committee of the First Hospital of Jilin University (approval number 2021059, Changchun, China) and were carried out according to the relevant institutional guidelines and laws.

We agree to participate in this research by wearing the sensors to detect physiological pressure signals and electrophysiological signals.

Yang Guo

Jingwei Wei

xiangjiao Xia

Yizhou Zhao

Xie Li zhang

August 21st, 2021

Animal Ethics Statement

The animal tests were approved by the Institutional Animal Care and Use Committee of the First Hospital of Jilin University (approval number 20210872, Changchun, China) and were carried out according to the relevant institutional guidelines and laws. For the experiment, 36 adult female Sprague-Dawley (SD) rats (~220 g) were provided by Liaoning Changsheng Biotechnology Co. Ltd (Liaoning, China). All rats could drink water and eat food freely during the experiment period. The rats were fed in the laboratory for 1 week before operation to allow them to adapt to the new environment. All surgery procedures were performed under aseptic conditions.

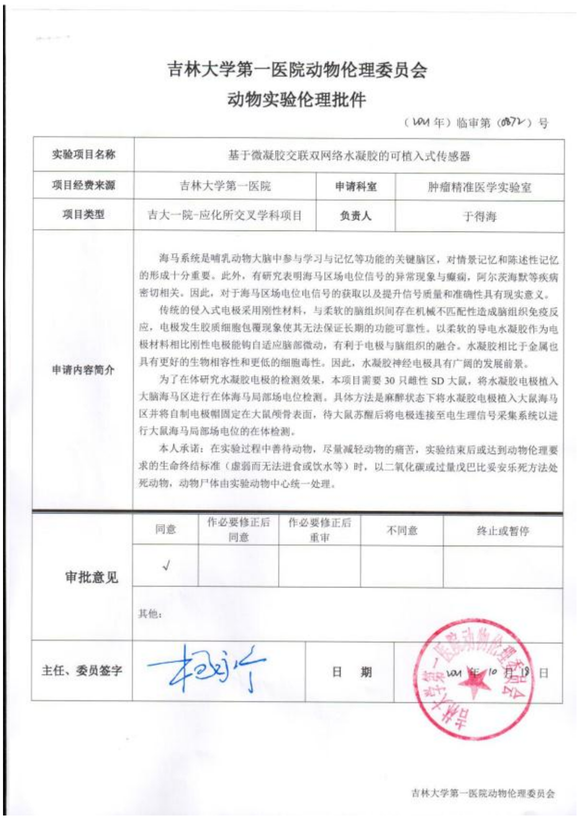
吉林大学第一医院动物伦理委员会
动物实验伦理批件

（2021 年）临审第（0872）号

实验项目名称	基于微凝胶交联双网络水凝胶的可植入式传感器		
项目经费来源	吉林大学第一医院	申请科室	肿瘤精准医学实验室
项目类型	吉大一院-应化所交叉学科项目	负责人	于得海
申请内容简介	海马系统是哺乳动物大脑中参与学习与记忆等功能的关键脑区，对情景记忆和陈述性记忆的形成十分重要。此外，有研究表明海马区场电位信号的异常现象与癫痫，阿尔茨海默等疾病密切相关。因此，对于海马区场电位电信号的获取以及提升信号质量和准确性具有现实意义。<br>传统的侵入式电极采用刚性材料，与柔软的脑组织间存在机械不匹配性造成脑组织免疫反应，电极发生胶质细胞包覆现象使其无法保证长期的功能可靠性。以柔软的导电水凝胶作为电极材料相比刚性电极能够自适应脑部微动，有利于电极与脑组织的融合。水凝胶相比于金属也具有更好的生物相容性和更低的细胞毒性。因此，水凝胶神经电极具有广阔的发展前景。<br>为了在体研究水凝胶电极的检测效果，本项目需要 30 只雌性 SD 大鼠，将水凝胶电极植入大脑海马区进行在体海马局部场电位检测。具体方法是麻醉状态下将水凝胶电极植入大鼠海马区并将自制电极帽固定在大鼠颅骨表面，待大鼠苏醒后将电极连接至电生理信号采集系统以进行大鼠海马局部场电位的在体检测。<br>本人承诺：在实验过程中善待动物，尽量减轻动物的痛苦，实验结束后或达到动物伦理要求的生命终结标准（虚弱而无法进食或饮水等）时，以二氧化碳或过量戊巴比妥安乐死方法处死动物，动物尸体由实验动物中心统一处理。		

审批意见	同意	作必要修正后同意	作必要修正后重审	不同意	终止或暂停
	√				
	其他：				

主任、委员签字	[signature]	日　期	2021 年 10 月 19 日

吉林大学第一医院动物伦理委员会

Implantation of the HM-2 electrode

SD rats were anesthetized with 2% isoflurane and fixed on a rat brain stereotaxic device (Shanghai Yuyan Instruments Co., Ltd.). The head skin was cut with surgical scissors to expose the skull. A skull drill was used to drill a hole where the electrode was placed, then a

hole on the opposite side was drilled and screwed in a stainless-steel screw to connect the ground wire. Subsequently, the HM-2 electrode was slowly inserted into the hippocampal CA1 area of the brain (AP-3.6mm, ML -2.0 mm, DV -2.7).

Recording LFPs of free-moving rats

After implanting the HM-2 electrode in the designated position, the part where the electrode was in contact with the skull was fixed with dental cement to prevent relative displacement. The prepared plastic cap was fixed on the skull with biomedical glue (3M Vetbond, 1469SB) to prevent the electrode from being damaged. Finally, the plastic cap was bonded with the terminal of electrodes together to make the whole device stronger. After the operation, the rat was placed in an electromagnetic shielding box with artificial light. After 48 hours of rest and adaptation to the environment, the rat’s LFP signals were acquired. The detective channel was connected to a preamplifier (SWF-1W, Chengdu Instrument Factory). The signals were amplified, acquired at a sampling frequency of 2 kHz, and filtered between 0.5 Hz and 300 Hz using an RM6240EC. Matlab 2018b software was used for signal processing.

Recording neuronal spikes of rats in vivo

The implantation method of the HM-2 microelectrode is the same as that of the HM-2 electrode described above. The HM-2 microelectrode was connected to a SWF-1W by a silver wire with a diameter of 300 μm. The signals were amplified, acquired at a sampling frequency of 40 kHz, and filtered between 500 Hz and 10 kHz using an RM6240E. The signals were threshold filtered to reduce noise. Matlab 2018b software was used for spikes sorting and signal processing.[3,4] The signal-to-noise ratio (SNR) was calculated by the formula:

$$\mathrm{GF} = 20\log_{10}\frac{V_\mathrm{s}}{V_\mathrm{n}} \qquad (2)$$

Where V_s and V_n are the mean value of the signal and noise voltages, respectively.

Immunohistochemistry and imaging of HM-2, platinum, and silver interfaces

In each rat tested, a hole was drilled in the skull and the implant electrodes (HM-2, platinum, or silver) were inserted in the hippocampus CA1 area (AP: −3.6 mm, ML: 2.0 mm, DV: −2.7 mm from bregma). After waiting for 1 week, the rats were perfused for the immunochemical experiment. Firstly, the mouse was deeply anesthetized with sodium pentobarbital (1% wt/vol) and was transcardially perfused with saline buffer followed by a fixative solution. Then the brain was placed at 4 ^{o}C for 6-8 h fixation. Next, the brain was dehydrated with sucrose (25% wt/vol) overnight. Finally, coronal cryosections were cut at 50 μm on a freezing microtome (Leica RM2016) for confocal imaging and immunostaining. After being gently rinsed with xylene (2 × 15 min), ethanol (2 × 5 min), 85% alcohol (5 min), 75% alcohol (5 min), and distilled water, the brain slices were incubated with one of the following primary antibodies: anti-GFAP (rabbit, 1:100, Proteintech Group), anti-Iba1 (rabbit, 1:300, Wako) at 4 °C overnight. The next day, the sections were rocked and washed 3 × 5 min by PBS buffer (pH = 7.4) and then incubated with fluorophore-conjugated secondary antibody for 50 min at room temperature (1:400: Yiyuan Biological Technology Co., Ltd.). Finally, all images were acquired with 20× objectives using a confocal microscope (Nikon Eclipse TI-SR).

Counting the number of glial cells and calculating mean fluorescence intensity.

The cell count refers to the reported method.[2] Three persons counted glial cells on each confocal image, and the median of the three counts was used to represent the image. To compare fluorescence intensity, confocal images were acquired under the same microscope parameter, and the fluorescence intensity was calculated by Image J under the same parameter.

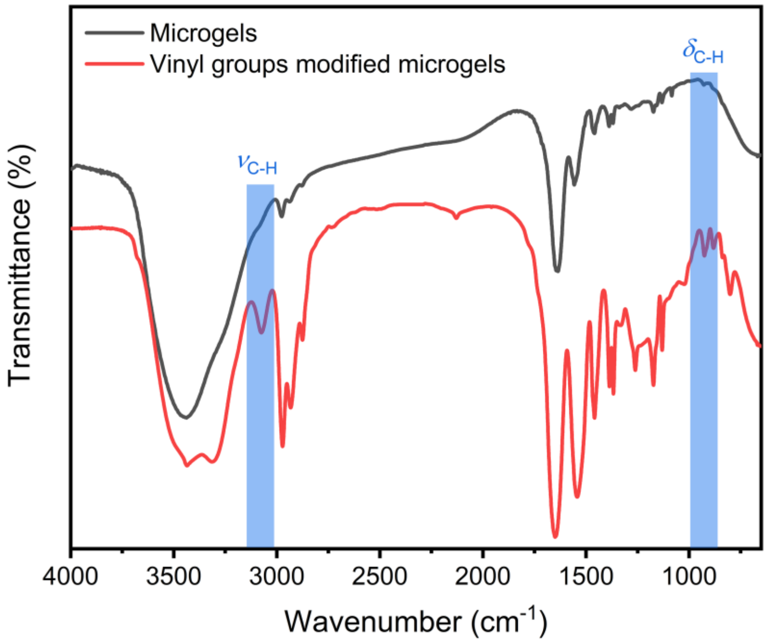

Figure S1. IR spectra of microgels.

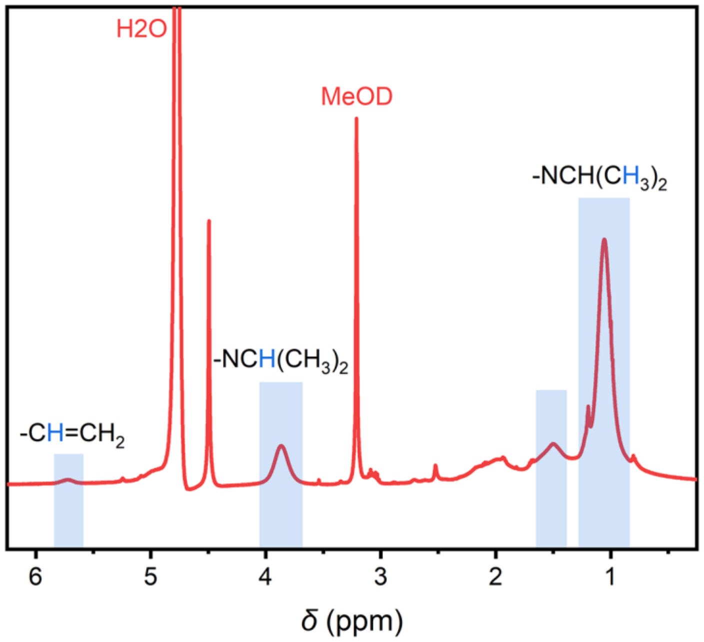

Figure S2. ^{1}H-NMR spectrum of the vinyl group modified microgels. The molar ratio of the actual involved 3-butenylamine hydrochloride and NIPAm in the reaction is calculated to be 1:9 according to the integral area of the characteristic peak (δ=5.79-5.67, -C**H**=CH_2; δ=4.21-3.56, -NC**H**(CH_3)$_2$).

Table S2. Influence of microgels contents on the mechanical performance of hydrogels.

Samples	Microgels content [wt% to AAm]	Elastic modulus [kPa]	Tensile strength [MPa]	Elongation at break [%]	Toughness [MJ/m^3]
HM-0	0.0	57±2	0.71±0.07	1400±50	3.3±0.3
HM-1	1.0	60±2	0.86±0.02	1660±30	5.1±0.6
HM-2	2.0	59±3	1.01±0.05	1870±20	6.6±0.2
HM-3	3.0	58±5	0.81±0.05	1840±30	5.2±0.1
HM-4	4.0	57±1	0.63±0.03	1880±10	4.6±0.2
HM-5	5.0	47±4	0.59±0.03	1890±50	3.8±0.6

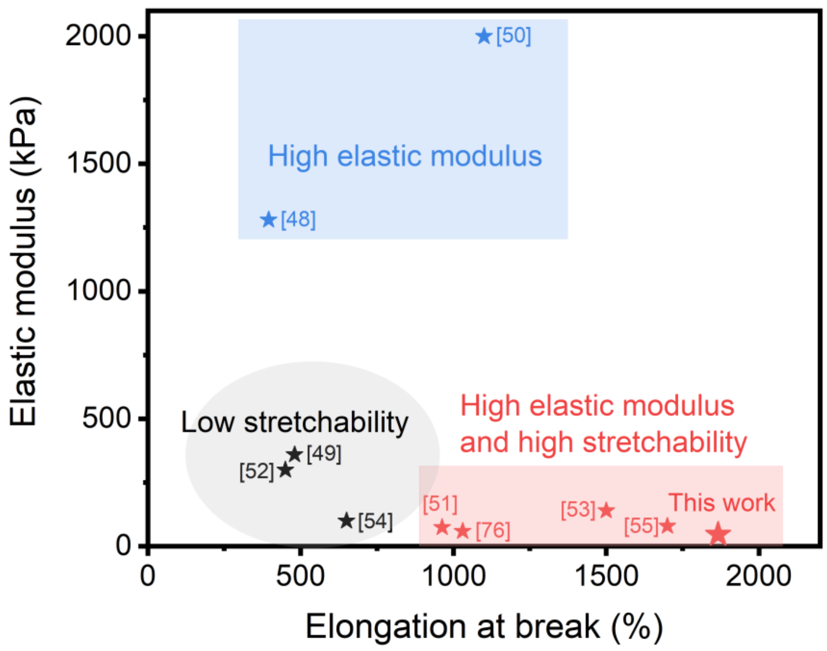

Figure S3. Comparison of HM-2 with other reported hydrogel strain sensors in terms of elongation of break and elastic modulus.

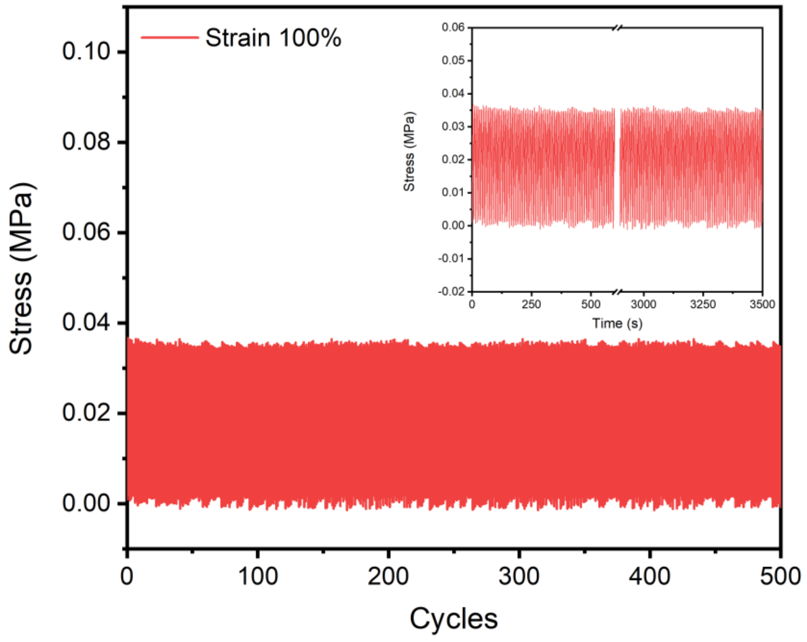

Figure S4. Stress changes of HM-2 sensor during 500 consecutive tensile cycles under 100% strain.

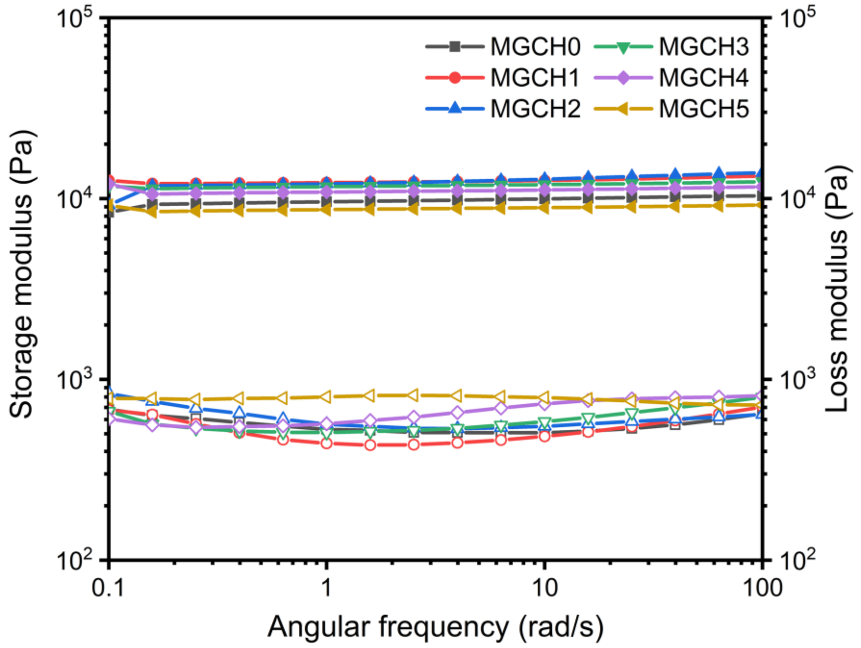

Figure S5. The storage modulus G' (solid symbols) and loss modulus G'' (hollow symbols) of the hydrogels as a function of frequency from 0.1 to 100 rad/s.

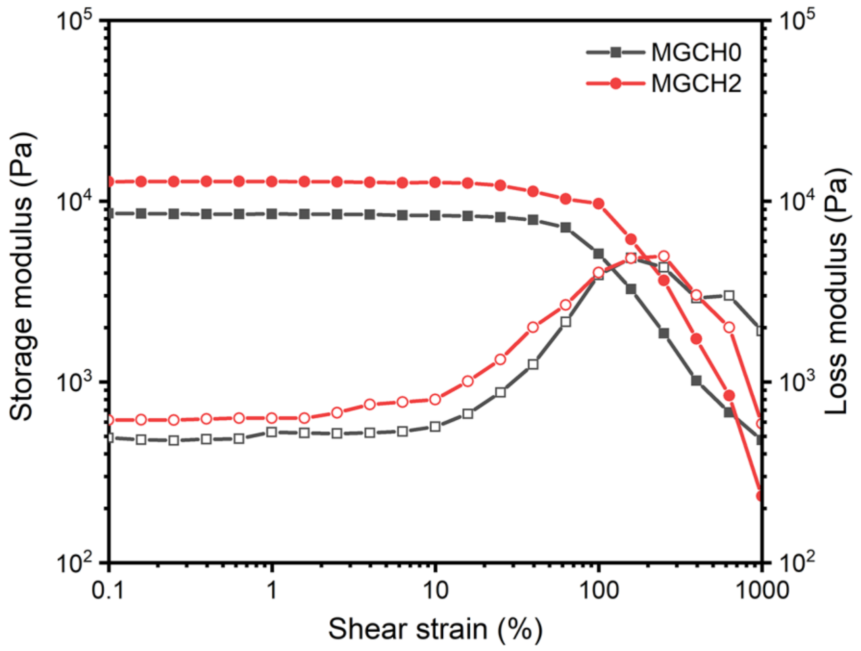

Figure S6. *G′* (solid symbols) and *G″* (hollow symbols) of the hydrogels as a function of shear strain from 0.01 to 1000%.

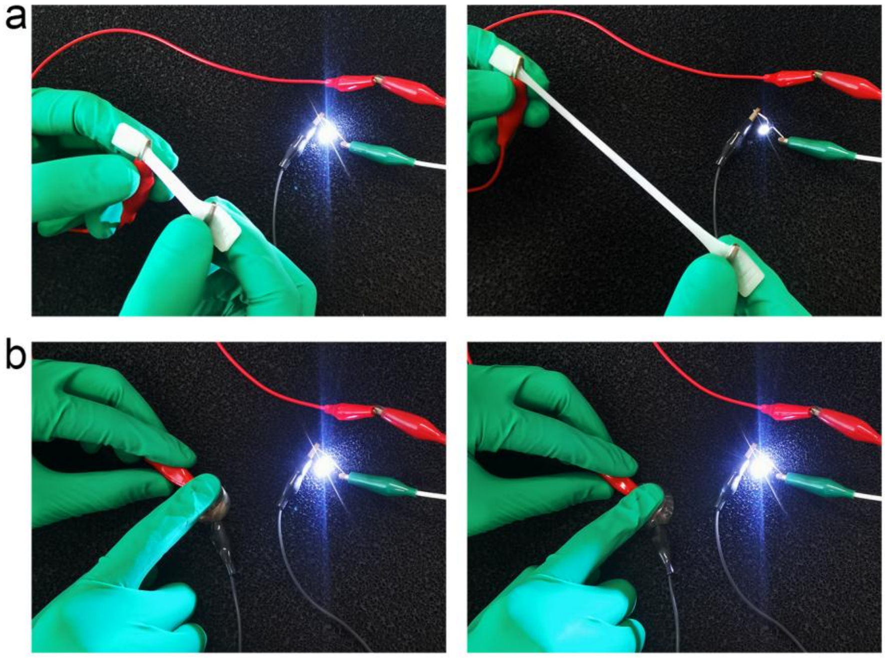

Figure S7. The brightness changes of the LED light when HM-2 was stretched (a) and pressed (b).

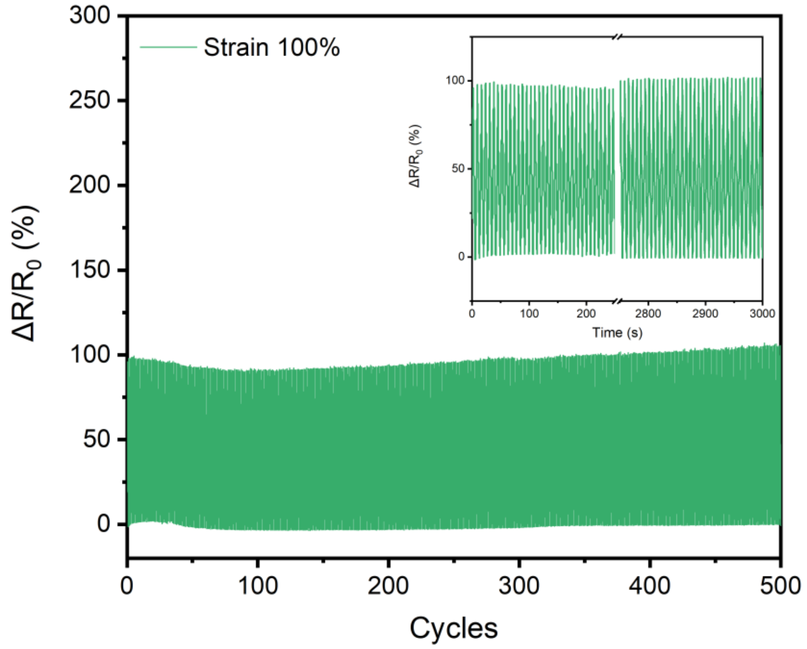

Figure S8. Resistance changes of a HM-2 sensor during 500 consecutive tensile cycles under 100% strain.

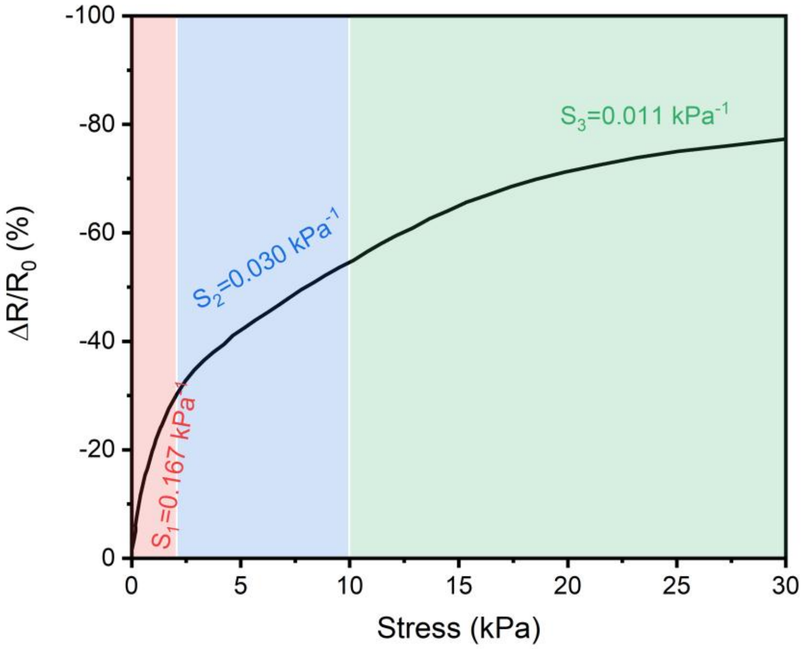

Figure S9. Resistance change-stress curve of a HM-2 pressure sensor.

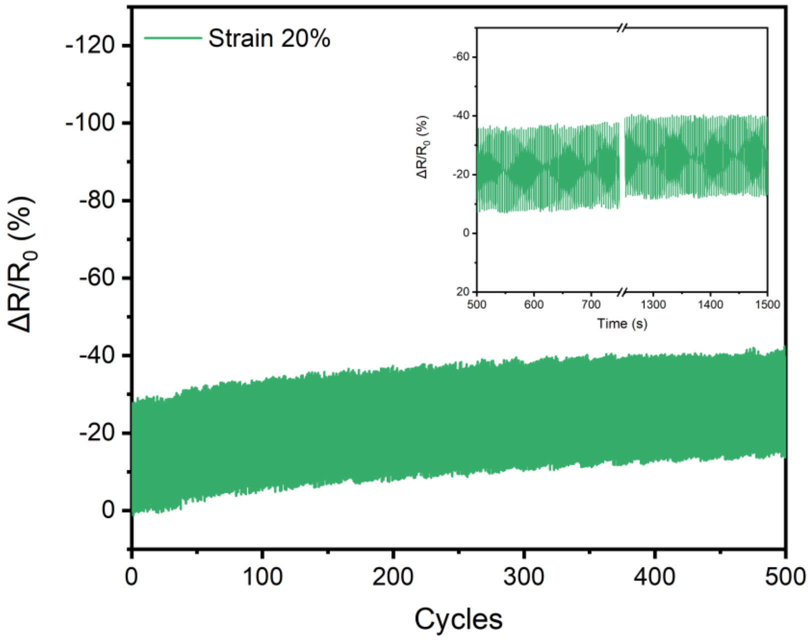

Figure S10. Resistance changes of a HM-2 sensor during 500 consecutive compression cycles under 20% strain.

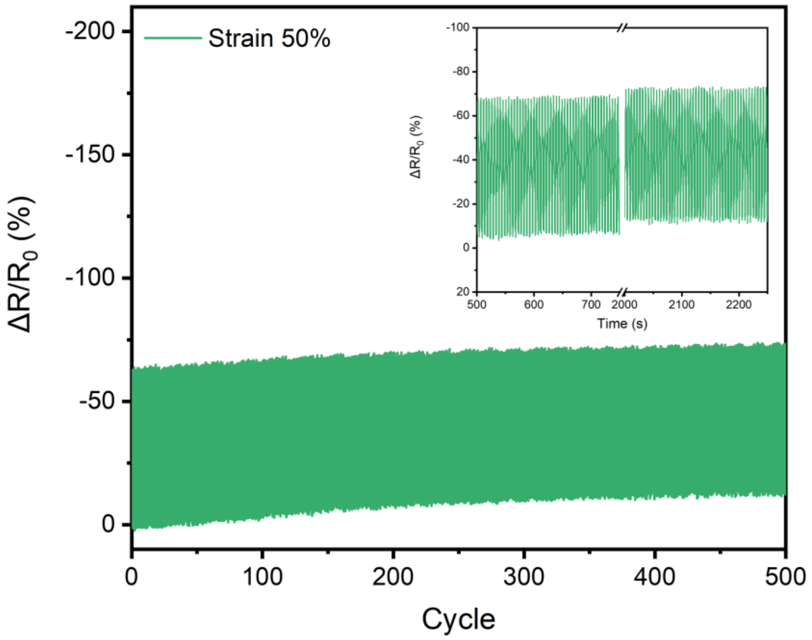

Figure S11. Resistance changes of a HM-2 sensor during 500 consecutive compression cycles under 50% strain.

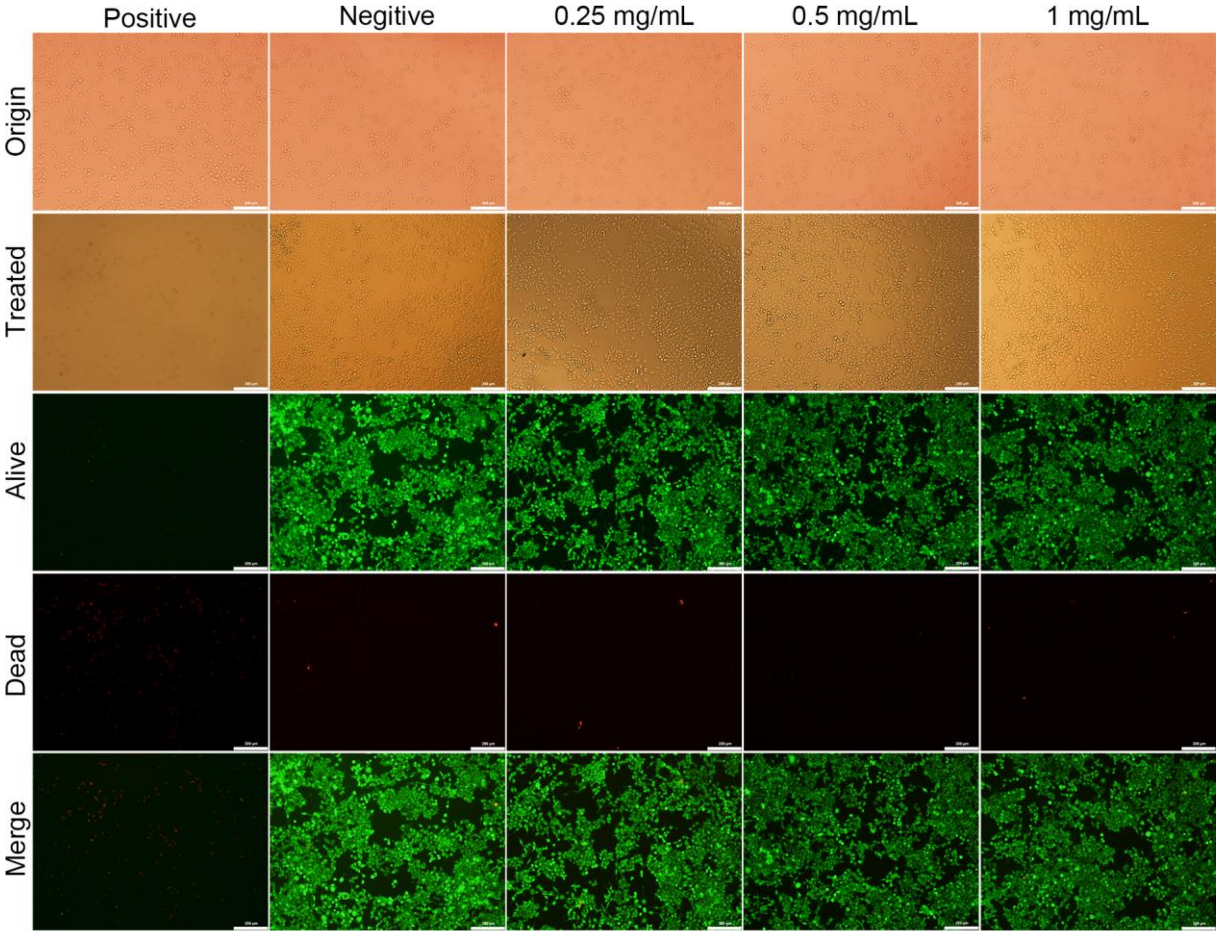

Figure S12. Photomicrographs (first two rows) and fluorescence photos (last three rows) of the cytotoxicity test of HM-2. Positive is the toxicity control group, and almost all the cells died. Negative is a non-toxic control group. Green fluorescence represents live cells, and red fluorescence represents dead cells.

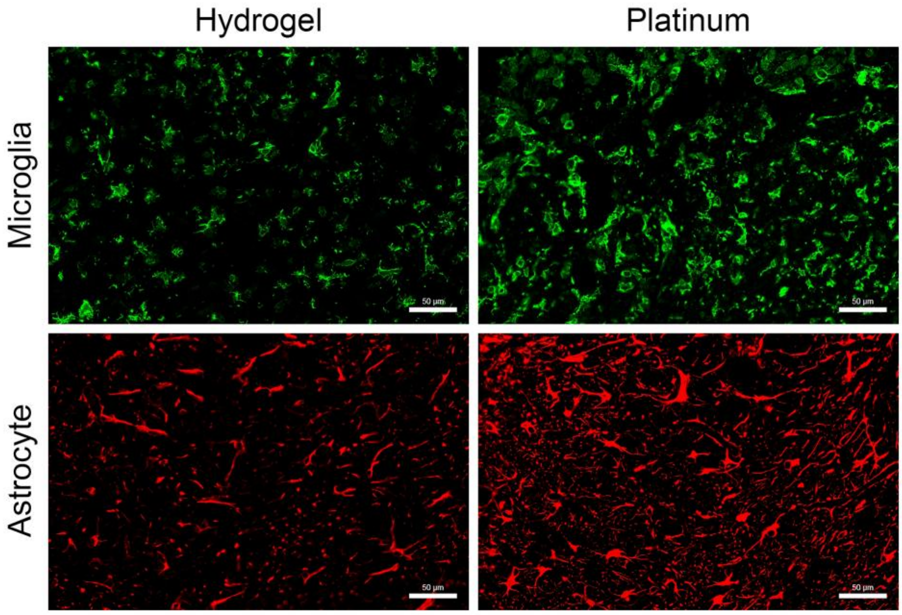

Figure S13. Confocal micrographs of the brain slice around the electrodes after 4 weeks of implantation in rats' brains: microglia cells (green) and astrocyte cells (red).

References

[1] C. D. Sorrell, M. J. Serpe, *Adv. Mater.* **2011**, *23*, 4088.
[2] H. Sheng, X. Wang, N. Kong, W. Xi, H. Yang, X. Wu, K. Wu, C. Li, J. Hu, J. Tang, J. Zhou, S. Duan, H. Wang, Z. Suo, *Extreme Mech. Lett.* **2019**, *30*, 100510.
